# Cardiovascular diseases in the mirror of science 

**DOI:** 10.15171/jcvtr.2016.32

**Published:** 2016-12-27

**Authors:** Mohammad-Hossein Biglu, Mostafa Ghavami, Sahar Biglu

**Affiliations:** ^1^Basic Sciences Department, Paramedical Faculty, Tabriz University of Medical Sciences, Tabriz, Iran; ^2^Radiology Department, Paramedical Faculty, Tabriz University of Medical Sciences, Tabriz, Iran; ^3^Asien-Afrika-Institute, Hamburg University, Hamburg, Germany

**Keywords:** Heart Disease, Cardiovascular Disease, Scientometrics, SCI-E

## Abstract

***Introduction:*** Heart disease or cardiovascular disease (CVD) is a kind of illness that involve heart and/or blood vessels of people throughout the world. The major aim of current study was to show the trend of global scientific activities in the field of CVD during a period of 10 years through 2001-2010.

***Methods:*** A scientometrics analysis was carried out to show the world wide activities towards scientific production in the field of CVD during a period of 10 years. Science Citation Index- Expanded (SCI-E) was used to extract all documents indexed as a topic of CVD throughout 2001- 2010.

***
Results:*** Analysis of data showed that the number of publications in the field of cardiovascular has increased steadily. The number of publication indexed in SCI-E in 2010 was three times greater than in 2001. It reached from 5080 documents in 2001 into 15,584 documents in 2010. English consisting 95% of total publication was the most dominant language of publications. Based on Bradford scatterings law the journal of Circulation was the most prolific journal among core journals. The USA sharing 29.5% of world’s profiles in the field was the most productive country Harvard University was the most productive Institution followed by Brigham Women’s Hospital.

***Conclusion:*** The vast majority of scientific publication in the field of CVD was produced by authors from North America and Western Europe. The results of study concluded that research activities in the field of CVD have become an interesting subject area of scientists during years 2001-2010.

## Introduction

The excessive changes in the lifestyles of inhabitants across the world through the last decades has moved the human societies from farming foods and active lives into fast foods and inactive lifestyles.^1^ The grouping of such lifestyle with growing cigarette consuming has improved the risk factors of cardiovascular diseases (CVDs).

Heart disease or CVD is a kind of illness that involve heart and/or blood vessels of people throughout the world. It is the main reason of deaths and ineffectiveness in the United States as well as in many European countries. The initial signs of CVD were described through a surgery performance on a soldier who was injured in a battle in Vietnam. According to the official reported the plaque was detected in the arteries of almost 90% of injured solders in Vietnam. It is remarkable to announce that all these troops were under twenty years old. This is a hint that it is crucial the CVD to be taken under emergency consideration, what causes it, and how the health care administrations should prevent it.^2^ based on the official reports almost 59,000 people was died because of CVD in New York state in 2007.^3^ The British Heart Foundation Statistics database has stressed that although the rate of deaths due to the CVD in the United Kingdom is decreasing, but the heart and cardiovascular illness is still the main reason of deaths in the United Kingdom, more than 191 000 persons die from CVD in UK annually. The cause of great number of deaths among men and women aged under 75 is still CVD.^4^ The WHO has emphasized that CVD is recognized as the chief reason of deaths throughout the world, it causes more than 30% of all deaths in the world. The CVD affects the people disproportionally in low- and middle-income countries. About 82% of deaths due to the CVD happens in low- and middle-income countries, the rate of death happens approximately similarly in men and women. It is predicted that by year 2030 about 23.6 million people will die from CVD annually. The biggest growth number of deaths will happen in the South-East Asia region.^5^ It is essential consideration for policy makers in countries to take into consideration the prevention of this disease. Not only the rate of mortality by this disease among people is high, but also it costs a lot of money and energy for treatment and surgery. Considering the direct health care expenditure, we find that the CVD seems to be the most expensive health state, it costs roughly 11% or 5.4 billion dollars from whole assigned health system expenditure in 2000-2001.^6^ It is estimated that the whole charge of CVD in 2008 in New York state was about $32.6 billion. On the other hand, many people who survive from CVD are disabled and cannot manage their lives productivity. According to the declaration of WHO the CVDs are the chief reasons of death and disability in the world. Based on the estimation nearly 17.3 million people died due to the CVDs in 2008. This rate demonstrated 30% of all worldwide deaths. More than 80% of CVD deaths happen in low- and middle-income countries.^7^ An epidemiologic study conducted in Tabriz University of Medical Sciences showed a high prevalence of risk factors among CVD patients; thus, urgent lifestyle modification was recommended.^8^ Director of Heart and Cardiovascular center of Iran’s Health Ministry emphasized that about 300 people die in Iran due to the CVD every day.^9^ Other study conducted by Akgün et al indicated that 35%-38% of deaths causing in Turkey includes major vascular illnesses.^10^ Analysis of published papers in subject areas is an appropriate indicator to show the scientists’ approaches towards desired subject areas. Descriptive study of scientific output in the field of CVD can reveal the involvement of scientists in the field. In addition, the study of published papers in subject area of CVD would show the link between their publication and research-interests; therefore, it is necessary to visualize the trend of scientific activities of CVD in order to be aware about the scientists’ approaches towards treatment and preventing the number one killer of patients worldwide. According to the information of Center for Disease Control and Prevention (CDC) on September 6, 2013, the whole rate of deaths has decreased about 29% related to the heart disease, hypertensive disease and stroke in the United States.^11^ This was an indication that the declination of death rate in CVD may correlated with the scientific activity in the field.

In this study we aimed to analyze and visualize the trend of scientific activities in the field of CVD by leading countries as reflected in their scientific output throughout 2001-2010.

## Materials and Methods

Science Citation Index Expanded (SCI-E) is a citation database that includes more than 8500 of the world’s important scientific journals across 150 disciplines from 1900 to the present. This database permits researchers to access the bibliographical-information of papers and to recognize which later papers have cited any specific previous article, or cited the articles of any particular author, or determine which articles have been cited most frequently. Science citation Index expanded (SCI-E) from database of web-of-science was used to extract all publication indexed as a topic of “*cardiovascular disease*” during a period of 10 years through 2001-2010. Based on the controlled vocabulary of Medical Subject Heading (MeSH), the term of “Cardiovascular disease” was selected as main keyword, for extracting data from the database of SCI-E. Our search strategy included all papers categorized as a topic of ‘‘cardiovascular diseases” in the SCI-E database. Limiting the extraction of data to the topic in SCI-E enable the researchers to find out the most appropriate papers in the preferred fields. After extraction of papers in the field of CVD, all paper checked manually for avoiding the duplication of papers. The extracted data imported into the soft-ware package of SPSS-22 for analyzing. Microsoft Excel-16 was used to plot the charts.

## Results

**Figure 1:**
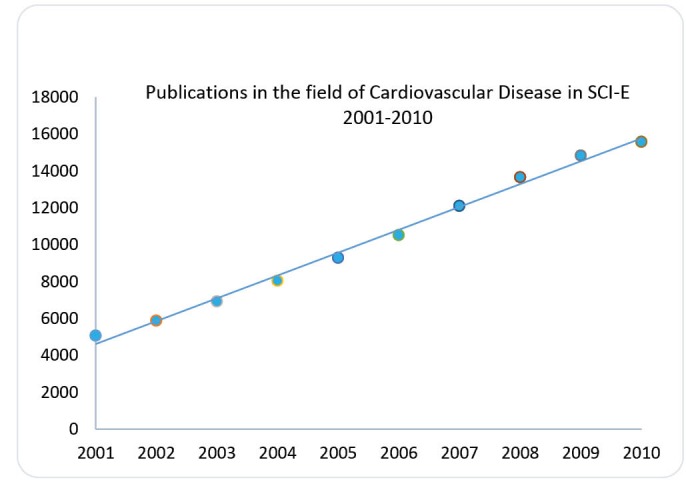
Number of publication in the field of cardiovascular disease in SCI-E 2001-2010.

Our search resulted a total number of 98143 documents in the form of article, editorial, book, biography, review, meeting, correction, other, abstract, letter, news, and bibliography. Analysis of data extracted from database of SCI-E indicated that the number of publication in the field of CVD has increased linear throughout 2001-2010 (Figure 1).

As Figure 1 indicates the number of publication has increased steady through the period of study. The number of publication in 2010 was three times than those in 2001.

**Figure 2:**
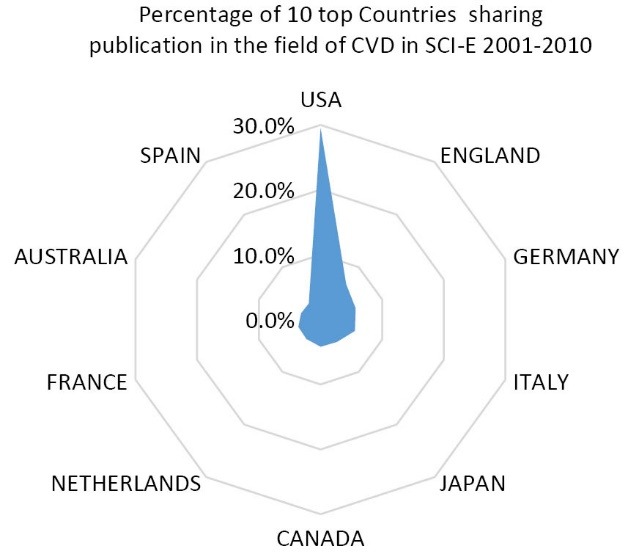
Distributing of scientific production by origin country of authors in the field of cardiovascular disease through 2001-2010.

Figure 2 shows the percentage of origin countries for authors sharing their publications in the field of CVD during the period of study. As figure shows the most majority of scientific profiles in the field came from North America and West Europa. USA contributing 29.5% of total scientific production is the most productive country followed by England (6.7%), Germany (5.7%), Italy (5.6%) and Japan (4.3%).

**Figure 3:**
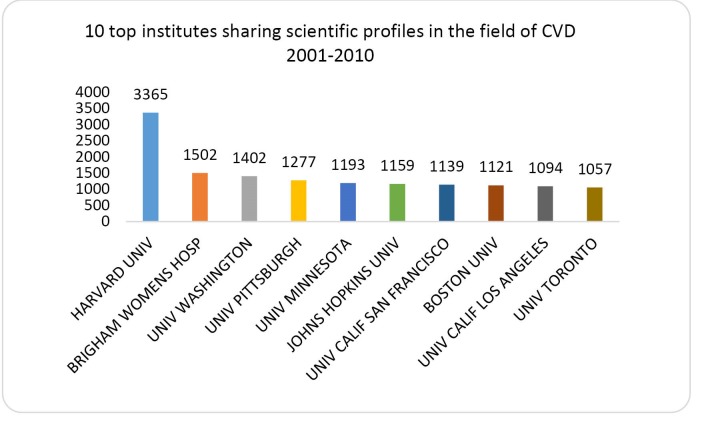
Ten top institutes sharing scientific production in the field of cardiovascular during 2001-2010.

Based on the data of Figure 3 Harvard University contributing of 3.3% of total production in the field is the most productive institution. The followings are Brigham Womens Hospital (1.5%), University of Washington (1.4%), University of Pittsburgh (1.3%), University of Minnesota (1.2%), Johns Hopkins University (1.1%), University of Calif San Francisco (1.1%), Boston University (1.1%), University of Calif Los Angeles (1.1%) and University of Toronto (1.0%). The figure is restricted to the countries that published greater than 1000 papers in the field.

English consisting 95% of total publication indexed in SCI-E throughout the period of study is the most dominant language of publication in the field of CVD followed by French, Spanish and German respectively. The language of 2% of total publication stayed unspecified (Table 1).

**Table 1:** Frequency of languages for publication in the field of CVD 2001-2010

**Language**	**Publication**	**Percent**
English	96 895	95
Unspecified	1735	2
French	1048	1
Spanish	701	1
German	672	1
Portuguese	365	0
Russian	266	0
Polish	78	0
Italian	69	0
Japanese	44	0
Korean	39	0
Turkish	31	0
Czech	17	0
Serbian	6	0
Dutch	5	0
Slovenian	4	0
Croatian	3	0
Romanian	2	0
Slovak	2	0
Chinese	2	0
Hungarian	2	0
Danish	1	0
Estonian	1	0
Rumanian	1	0
Serbo Croatian	1	0
Total	101 990	100

As Table 2 indicates the vast majority of publication (71.3%) was in the form of journal articles followed by review (14.2%) and proceedings papers (5.7%).

**Table 2:** Frequency of publication types for publication in the field of CVD 2001-2010

**Publication Type**	**Frequency**	**Percent**
Article	77224	71.3
Review	15361	14.2
Proceedings paper	6193	5.7
Editorial material	4288	4.0
Meeting abstract	3555	3.3
Letter	1197	1.1
Book chapter	178	0.2
Correction	167	0.2
News item	112	0.1
Reprint	50	0.0
Biographical item	16	0.0
Book review	9	0.0
Bibliography	3	0.0
Hardware review	1	0.0
Meeting summary	1	0.0
Total	108355	100.0

Table 3 shows the list of journals that published the scientific papers in the field of CVD. The table is restricted to the journals name that published greater than 500 papers in the field. From 3,075 kinds of journals that contributed the publications in the field of CVD, the journal of “*Circulation”* with publishing 2.70% (2,741 papers) of total scientific papers in the field was the most productive one followed by “*Atherosclerosis*” 1.59% (1616 paper), “*American Journal of Cardiology*” 1.29% (1,311 papers), *Journal of The American College Of Cardiology* 1.26% (1279 papers), *Journal of Hypertension* 1.15% (1170 papers), and *Diabetes Care* 1.00% (1021 Papers).

**Table 3:** Frequency of journals published scientific papers in the field of CVD 2001-2010

**Journal Name**	**No.**	**Percent**
Circulation	2741	2.70
Atherosclerosis	1616	1.59
American Journal of cardiology	1311	1.29
Journal of the American College of Cardiology	1279	1.26
Journal of Hypertension	1170	1.15
Diabetes Care	1021	1.00
European Heart Journal	947	0.93
Hypertension	835	0.82
International Journal of Cardiology	825	0.81
Nephrology Dialysis Transplantation	788	0.78
Arteriosclerosis Thrombosis and Vascular Biology	783	0.77
American Journal of Clinical Nutrition	779	0.77
American Heart Journal	764	0.75
Kidney International	745	0.73
Journal of Clinical Endocrinology Metabolism	695	0.68
Stroke	645	0.63
American Journal of Hypertension	620	0.61
Archives of Internal Medicine	602	0.59
American Journal of Kidney Diseases	583	0.57
JAMA	540	0.53
Journal of The American Society of Nephrology	520	0.51
Metabolism Clinical and Experimental	520	0.51
American Journal of Epidemiology	518	0.51

## Discussion

Analysis of obtained data from database of SCI-E indicated that the number of publication in the field of CVD has experienced continues growth; so that the number of publication in 2010 stands 207% greater than those in 2001. It reached from 5080 documents in 2001 into 15,584 in 2010. Considering analysis of linear regression (Figure 1) indicates a significant relationship between the number of papers and the years of under investigation (*P* ≤ 0.001). Due to the fact that aaccurate recognition of scientific activities is suitable indicator for revealing the trend of science and technology in institutions and/or countries which may be used as statistics that evaluate the measureable features of the structure, spreading and application of science and technology.^12^ This mechanism is essential to empower the researchers to plan their research projects and the institutions to progress their research systems efficiently; therefore, the increasing trend of scientific output in the field of CVD can be interpreted in a way that research activities in the field of CVDs have become an interesting issue among cardiovascular scientists and/or institutions since last decade. Among 10 top countries the North America (USA and Canada) sharing 33.6% of world publication was the most productive region followed by Western Europe. This study is not the first that indications the technical impacts of these regions in the world; other previous surveys specified the major role of these regions in science too.^13-15^ The results of our study are in consistence with the results of a recent cardiovascular bibliometric analysis which showed nearly 36% growth in CVD publications during the last 10 years.^16^

Prabhakaran et al showed also a similar distribution of cardiovascular scientific profiles from high-income countries regarding to the publication analysis which they extracted from Medline database during years 1994–1995 and 2004–2005.^17^ Their study showed that high-, upper middle-, lower middle-, and low-income countries distributed 82%, 7%, 7%, and 4% of publication in cardiovascular fields, although their study covered only 90 countries. In other study Mendis et al discovered a similar amount of publication in the field of cardiovascular from developed-market-economies in a limited search MEDLINE in 1991 (78%), 1996 (79%), and 2001 (78%).^18^ The probability reason of this dissimilarities may be due to different search plans and absence of collaborative filters test provided by Prabhakaran et al and Mendis et al.

McKee et al proposed that the lack of governmental or non-governmental outcomes and the lack of health research plan, absence of suitable resolution provided by policy-makers may lead to prioritize health research, geographic separation, and current engagement.^19^ Some studies stated that the rising dominance of English language in scientific papers may be one obstacle for improvement of researches in the field of CVDs, mainly in low- and middle-income countries.^20^

This noticeable point seems to be because of the deadliness of CVD in the regions. At present the heart disease is the main cause of deaths among men and women. It is remarkable to know that the cardio vascular disease threatens the children as well and may affect them even in the womb if the mother’s diet is inadequate through the pregnancy-periods.^21^ On the other hand, we are aware that the cost of CVD is very high. It is estimated that the total cost of cardiovascular in the United State was over $448.5 billion in 2008.^22^ The ten top productive countries that shared 69.3% of global publications were: The USA sharing 37 823 papers (29.5%), England 8643 (6.7%), Germany 7263 (5.7%), Italy 7152 papers (5.6%), Japan 5476 papers (4.3%), Canada 5339 papers (4.2%), Netherlands 4714 papers (3.7%), France, 607 papers (3.6%), Australia 4017 papers (3.1%) and Spain 3922 papers (3.1%). Noticeably Japan from Asian region and Australia from ocean region hold the fifth and 10th ranks among ten top productive countries, consisting of 5476 and 4017 papers with global publication share of 4.3% and 3.1% respectively. Our findings are in agreement with the findings of Jahangir et al who reported that Latin America published 4% of CVD publications compared with 26% from the USA with a slow upward trend over time.^23^ Regarding to the institutes, Harvard University sharing 3.3% of world’s publication holds the first rank, the followings are Brigham Women’s Hospital (1.5%), University of Washington (1.4%), University of Pittsburgh (1.3%), University of Minnesota (1.2%), Johns Hopkins University (1.1%), University of California**--**San Francisco (1.1%), Boston University (1.1%), University of California**--**Los Angeles (1.1%) and University of Toronto (1.0%). 71.3% publication was in the form of Journal articles followed by Review (14.2%) and proceedings Papers (5.7%). English language consisting 95% of total publication indexed in SCI-E throughout the period of study was the dominant language of papers in the field of CVD. The dominance of English language for papers extracted from this database should not come as a wonder, the editorial policy of American bibliographical databases such as MEDLINE and Web of Science has focused indexing the papers in English language.^24^ A great number of CVDs is preventable by warning the people about the behavioral risk factors such as heavy smoking, unhealthy food diet, excessive obesity, physical inactivity and consuming of alcohol.^25^

## Conclusion

The results of study concluded that although the research activities in the field of CVD have become an interesting subject area of scientists, so that the number of published papers in the field has grown linear through the last decade, but considering the high incidence of diseases related to the cardiovascular, these activities is not good-enough.

The practical implications of CVD are high importance. The policy makers in the countries should bear in mind that People with high risks of CVD need timely determination and appropriate counselling and medicines.

## Limitations of the study

This study focuses solely on the papers indexed as a topic of “cardiovascular disease” in the database of SCI-E, it has some limitations. We are aware that, there is no comprehensive database to mirror all publications in the field of CVD from all countries, for this reason we had to select a database which can show the majority of scientific output in the field. For this reason, we selected the SCI-E database.

There are more publications in the field of CVD in languages other than English within the countries, which are not included in the SCI-E. Our recommendation for further studies is to take these publications into consideration in the future studies.

## Ethical approval

None to be declared

## Competing interests

The authors declare no competing interests.

## Acknowledgments

We are very grateful for all people who helped us through accomplishing this study.
